# Advancing CAR-T therapy in multiple myeloma: a critical appraisal of CARTITUDE-4 and the path toward earlier lines

**DOI:** 10.3389/fonc.2026.1811170

**Published:** 2026-05-26

**Authors:** Chao-qun Zhou, Shi-Qiong Zhou, Qing-Hua Ke

**Affiliations:** Department of Chemoradiotherapy, Jingzhou No1 People’s Hospital and First Affiliated Hospital of Yangtze University, Jingzhou, Hubei, China

**Keywords:** CAR T-cell therapy, CARTITUDE-4 trial, cilta-cel, health equity, multiple myeloma, overall survival

## Introduction

The updated overall survival (OS) analysis from the phase 3 CARTITUDE-4 trial represents a watershed moment, demonstrating that a single infusion of the BCMA-directed CAR T-cell therapy ciltacabtagene autoleucel (cilta-cel) significantly improves OS compared to standard care in lenalidomide-refractory multiple myeloma after 1–3 prior lines (1). As the first phase 3 CAR-T trial in myeloma to show a statistically significant OS benefit, it solidifies cilta-cel as a transformative option and compels the field to confront pressing questions about moving CAR-T earlier, alongside implications for trial design, safety, and equity.

## Core contributions and clinical value

The trial’s paramount contribution is confirming a significant OS advantage (HR 0.55, p=0.0009) at 33.6 months median follow-up, building on its prior progression-free survival (PFS) benefit (2). The no-crossover design provides an uncontaminated survival assessment. High rates of minimal residual disease negativity (62% at 10^-5^) and delayed symptom worsening underscore that deep, durable responses translate into survival and quality-of-life gains. The data support considering cilta-cel as early as first relapse in lenalidomide-refractory patients, suggesting a potential shift from the paradigm of reserving CAR-T for later lines, while the current evidence does not yet mandate definitive practice change. Consistent benefit across most subgroups, including high-risk cytogenetics, strengthens its broad potential in earlier relapse.

## Critical evaluation and methodological considerations

Several limitations necessitate careful interpretation. The static control arm (PVd/DPd), while appropriate at design, may magnify cilta-cel’s benefit relative to newer regimens, underscoring the need for head-to-head trials against contemporary standards (3–5). Specifically, the control regimens (pomalidomide, bortezomib, dexamethasone or daratumumab, pomalidomide, dexamethasone) represent standard options at the time of trial design but have since been supplemented by novel combinations, potentially overestimating the relative benefit of cilta-cel. Early separation of PFS/OS curves highlights a critical, modifiable real-world factor: effective bridging therapy. However, the trial did not standardize bridging regimens, leaving uncertainty about how much of the early benefit is attributable to bridging versus cilta-cel itself; future protocols should prioritize standardized, potent bridging strategies.

The no-crossover design, while scientifically clean for OS estimation, creates ethical and practical tensions by denying post-progression access to cilta-cel for control-arm patients, emphasizing the balance between regulatory endpoints and patient access within trials. Although this design provides an unbiased estimate of OS, it raises ethical concerns regarding patient access to a potentially life-prolonging therapy after progression, highlighting a tension between rigorous endpoint assessment and patient welfare.

Safety signals demand long-term vigilance. While grade 3–4 cytopenias were common, reports of secondary hematological malignancies, including CAR-positive T-cell lymphoma, warrant careful monitoring (6). However, the absolute risk appears low based on current follow-up, and causality remains uncertain given patients’ prior exposure to multiple lines of therapy. Potential mechanisms range from insertional mutagenesis to immune selection pressure (7). This risk necessitates lifetime monitoring guidelines, explicit informed consent, and translational research to identify predisposing factors, aligning with recent regulatory communications. Toxicity assessment per consensus grading (8).

Generalizability and equity gaps exist. The trial population (good ECOG status, organ function) may not reflect older, frailer patients with comorbidities seen in practice. Lack of racial and socioeconomic diversity limits understanding of how these factors influence outcomes. Given that CAR-T access is shaped by cost, geography, and systemic biases, the findings apply most directly to a subset, highlighting significant implementation challenges.

## Interdisciplinary and systemic perspectives

Biologically, earlier CAR-T use may capitalize on better T-cell fitness and a less immunosuppressive microenvironment, a hypothesis supported by lymphoma trends. Translational studies correlating pre-infusion T-cell phenotypes and microenvironment signatures with outcomes are crucial for refining selection (7, 9, 10).

From a health systems perspective, logistical and financial challenges are monumental. The high upfront cost (>$400,000), as highlighted in recent cost-effectiveness analyses (11), need for specialized centers, and long-term monitoring create profound barriers; disparities in access by race, insurance, and geography have been documented (12). Innovative models are urgently needed, such as hub-and-spoke networks for expanded reach, investment in decentralized manufacturing to reduce costs/delays, and condition-based reimbursement tied to durable response. Without systemic innovation, earlier CAR-T will exacerbate global oncology inequities.

Cross-disciplinary insight from solid tumors is informative. The shift of immune checkpoint inhibitors into earlier lines in uro-oncology, grappling with similar cost, biomarker, and toxicity questions, offers lessons. Biomarker development (e.g., PD-L1, MSI) has been key for optimizing checkpoint inhibitor sequencing; similar pragmatic biomarker development is needed for BCMA-directed therapies in myeloma.

## Future directions and conclusions

CARTITUDE-4 mandates critical next steps: prospective trials directly comparing cilta-cel with contemporary standards; exploring efficacy in newly diagnosed high-risk myeloma; and non-negotiable biomarker development to identify patients with the optimal benefit-risk ratio. Real-world evidence must capture data from older, diverse, and comorbid populations.

On a policy level, multidisciplinary collaboration is required to streamline manufacturing, develop sustainable financing, and create equitable access pathways. Guidelines must evolve to include CAR-T as an early option, coupled with investments in infrastructure and training for toxicity management and long-term follow-up.

In conclusion, CARTITUDE-4 is a landmark trial demonstrating that CAR-T can significantly prolong survival when used earlier in the relapsed/refractory continuum, providing evidence to advocate for considering a paradigm shift rather than mandating an immediate change in practice (1, 2). However, translating this evidence into equitable practice hinges on overcoming substantial hurdles: optimizing patient selection and bridging therapy, mitigating long-term risks like secondary malignancies, and addressing systemic cost and access barriers. Realizing the promise of cellular immunotherapy for all patients requires rigorous science, honest dialogue on resource allocation, and compassionate policymaking.
